# Robot DE NIRO: A Human-Centered, Autonomous, Mobile Research Platform for Cognitively-Enhanced Manipulation

**DOI:** 10.3389/frobt.2020.00066

**Published:** 2020-07-23

**Authors:** Fabian Falck, Sagar Doshi, Marion Tormento, Gor Nersisyan, Nico Smuts, John Lingi, Kim Rants, Roni Permana Saputra, Ke Wang, Petar Kormushev

**Affiliations:** ^1^Robot Intelligence Lab, Imperial College London, London, United Kingdom; ^2^Department of Computing, Imperial College London, London, United Kingdom; ^3^Department of Bioengineering, Imperial College London, London, United Kingdom; ^4^Dyson School of Design Engineering, Imperial College London, London, United Kingdom; ^5^Research Center for Electrical Power and Mechatronics, Indonesian Institute of Sciences - LIPI, Jakarta, Indonesia

**Keywords:** mobile manipulation, perception for grasping and manipulation, manipulation planning, cognition-enabled manipulation, motion planning and control, dual-arm manipulation, human-centered manipulation, humanoid robotics

## Abstract

We introduce *Robot DE NIRO*, an autonomous, collaborative, humanoid robot for mobile manipulation. We built DE NIRO to perform a wide variety of manipulation behaviors, with a focus on pick-and-place tasks. DE NIRO is designed to be used in a domestic environment, especially in support of caregivers working with the elderly. Given this design focus, DE NIRO can interact naturally, reliably, and safely with humans, autonomously navigate through environments on command, intelligently retrieve or move target objects, and avoid collisions efficiently. We describe DE NIRO's hardware and software, including an extensive vision sensor suite of 2D and 3D LIDARs, a depth camera, and a 360-degree camera rig; two types of custom grippers; and a custom-built exoskeleton called DE VITO. We demonstrate DE NIRO's manipulation capabilities in three illustrative challenges: First, we have DE NIRO perform a fetch-an-object challenge. Next, we add more cognition to DE NIRO's object recognition and grasping abilities, confronting it with small objects of unknown shape. Finally, we extend DE NIRO's capabilities into dual-arm manipulation of larger objects. We put particular emphasis on the features that enable DE NIRO to interact safely and naturally with humans. Our contribution is in sharing how a humanoid robot with complex capabilities can be designed and built quickly with off-the-shelf hardware and open-source software. Supplementary Material including our code, a documentation, videos and the CAD models of several hardware parts are openly available at https://www.imperial.ac.uk/robot-intelligence/software/.

## 1. Introduction

Over the last decade, robots have increasingly appeared outside of factories and in homes (Bekey, 2012; Fortunati et al., 2015). Unique demands of the household environment have led to an explosion in robot designs. Robots in the home often need to be autonomously mobile and able to interact with dynamic environments and objects of unexpected shapes and sizes. Manufacturing robots, by contrast, are often rooted to one place, repeatedly performing a precise action. Domestic robots are frequently single-purpose or physically small (Noonan et al., 1993; Forlizzi and DiSalvo, 2006), though some do attempt to meet a broader set of needs (Hans et al., 2002). We propose a robot system whose design in both hardware and software goes in the latter direction: Robot DE NIRO (Design Engineering's Natural Interaction RObot).

**Figure 1:**
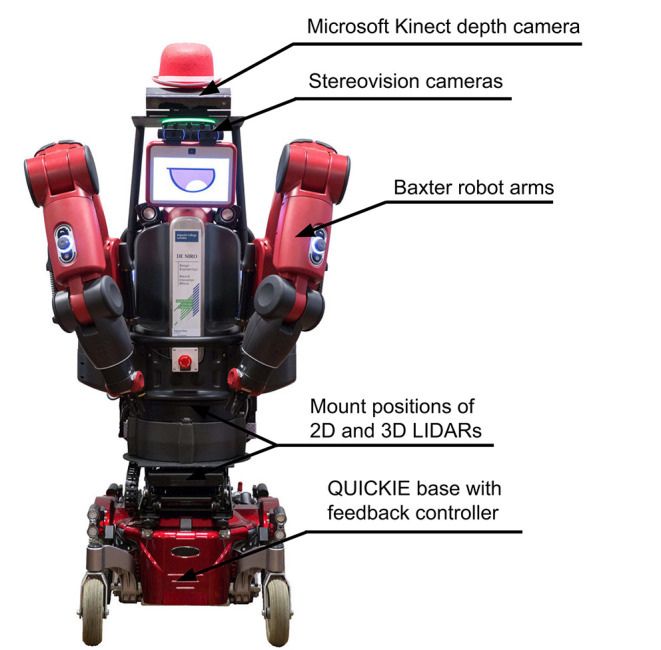
Robot DE NIRO—a collaborative research platform for mobile manipulation. The figure shows its design, main components, and sensors.

DE NIRO is a human-sized—and roughly human-shaped—robot whose most prominent features are its two manufacturing-grade arms. We depict DE NIRO in Figure 1. With its wide array of sensors, the extra mobility that comes from using a wheelchair base, and the software architecture that links all its parts together, DE NIRO can perform many tasks in dynamic environments, including object recognition, face recognition, and collision avoidance. We intend for DE NIRO to be a fully open-source and open-hardware research platform, and we have shared our source code and documentation, and videos of the robot in action at https://www.imperial.ac.uk/robot-intelligence/software/ (Robot Intelligence Lab, 2019).

We believe DE NIRO could be of particular use as an assistant to caregivers who are supporting the elderly. The abilities we have developed in DE NIRO may also be valuable more broadly, but we maintained caregiver assistance in a domestic environment as our guiding purpose. The focus of our research was therefore on building and designing DE NIRO in order to engage with human partners naturally, safely, and productively.

The overall aim of this paper is to introduce DE NIRO and its capabilities in detail, explaining how it was built, and what abilities we have built into it over three demonstrative challenges. However, our hope is also share this as a model for other researchers who may be interested in swiftly constructing their own robots with general capacities for natural interaction. We devote most of our space to describing the logic of how we approached certain problems, in order to allow other researchers to compare and perhaps apply their own solutions.

Key phrases or descriptors in robotics can be overlapping or ambiguous. For clarity's sake, we will define how we use certain terms in this paper.

**Term 1.1. *Automatic*** *describes an action (usually physical) that occurs or repeats independent of other stimuli*.

**Term 1.2. *Autonomous*** *describes an action (usually physical) that proceeds toward a goal, reacting to and working with external stimuli without human intervention*.

**Term 1.3. *Cognitive*** *describes the process of acquiring knowledge and making a choice or an action based on that knowledge. Acquiring knowledge usually requires some ability to perceive from the world, and a behavioral adjustment from that knowledge usually requires some ability to act on the world*.

**Term 1.4. *Intelligent*** *describes the ability to take in information from an environment, learn general principles, reason about their application, and then use that new knowledge to solve problems in new environments*.

In section 2, below, we review related work across robotics, with an emphasis on cognition-enabled manipulation and social assistance robotics. Subsequently, we present Robot DE NIRO in more detail, covering its sensors, actuators, and software in section 3.

Our three implementation sections begin with section 4, where we discuss how DE NIRO implements the classic pick-and-place task—in which a robot collects an object from one position and moves it to another. This task is a common challenge for industrial robots, which often collect, position, and assemble heavy parts (Engelberger, 2012). These applications do not require intelligence: the robots are placed in a highly controlled environment, where each motion is fully predefined and repeated. Industrial robots usually only move from one point to another without receiving or acting upon any information about their environments (Brogårdh, 2007). Thus, even though these solutions can be considered automatic, they cannot be qualified as autonomous.

Unlike these robots, DE NIRO does take in and respond to information from its environment in order to accomplish its goal without human interference. In doing so, it operates autonomously. This is particularly true when it attempts to navigate, collision-free, through a space, and as it adjusts its journey plan in response to new stimuli. In our later challenges, we focus more on the challenge of intelligence, giving DE NIRO a general principle to be applied to novel situations. Section 5 adds to DE NIRO's visual perception abilities, and section 6 adds to DE NIRO's grasping abilities. We conclude in section 7 by discussing DE NIRO's limitations and avenues for future work.

## 2. Related Work

In this section, we discuss previous work related to movement, manipulation, learning, and social assistance robotics.

### 2.1. Work on Movement

Recent years have seen the rise of many high-performing sensors that allow robots to perceive their environments far more accurately. The 3D LIDAR or the Microsoft Kinect can return a 3D point cloud of their surroundings by measuring the duration of time a ray of light takes to travel from the sensor to a surface and back again. DE NIRO employs both during navigation. Moving robots require collision maps to distinguish free and occupied zones. Those maps need to dynamically update and expand while the robot moves in order for it to avoid unexpected obstacles. Hornung et al. (2013) developed a solution called OctoMap to efficiently build and interpret 3D point clouds. Their algorithm uses probabilistic methods and octrees to efficiently map and to eliminate outliers in an evolving environment.

Zanchettin and Rocco (2013) discussed how to improve real-time motion planning. They hoped to simplify motion planning by applying the correct constraints. Their innovation was to have the robot solve its optimization problem in two “horizons”:

- The control horizon, taking into account sensor feedback to compose instantaneous values, like the configuration of a room at a particular instant and
- The planning horizon, composed of all non-instantaneous constraints (such as goal positions).

After several years of further work, Zanchettin and Rocco (2017) presented a full possible solution to the problem of simultaneous localization and mapping. Their dynamic motion planning technique regenerates a new path at every step in time. This algorithm is specifically relevant when a sudden external force is applied to the robot, which may force it to adapt its pathway immediately. In both papers, the authors evaluated the efficiency of their motion planning algorithm using an image-based grasping task.

### 2.2. Work on Manipulation

Grasping poses a large challenge to roboticists—specifically the process of planning a smooth, efficient arm movement to a target object and then enclosing that object securely. Rosell et al. (2011) tackled the issue of motion planning for anthropomorphic mechanical hands. They sought to map several human hand postures by first using a soft glove equipped with sensors, and then having a robotic hand reproduce those postures. In addition to that work, they detailed a well-commented principal motions directions algorithm for path-planning that is relevant to our study. Bagnell et al. (2012) were more driven by having a robot complete a variety of autonomous manipulation tasks. They built their own platform to support tasks that include drilling, unlocking doors, stapling papers, and more. Their RANdom SAmple Consensus (RANSAC) algorithm to identify and localize objects on a table is quite related to our work, especially to section 5, focused on object recognition and manipulation.

Feix et al. (2016) described a novel classification of 33 grasp types, the most complete classification to this day. The grasp types are separated by the need for power and precision during the grasp, but also by the size, weight, rigidity, and force requirements of the object being grasped. We can consider the robotic hands being used to have one thumb and two virtual fingers. Virtual fingers are opposing digits that can apply forces simultaneously in inverted directions. This taxonomy offers a vocabulary to build the testing set for possible grasp postures. It also allows us to elect the most suitable hand, depending on the dimensions of the object to be grasped. Vahrenkamp et al. (2010) focused on multi-robot motion planning, and in particular on dual-arm grasping. They present a motion planner for bimanual manipulation using RRT, and also some valuable points about inverse kinematics, which is relevant to our joint maneuvers.

To plan a grasping pose generically, the robot must have a clear understanding of the pose of target objects. Visual information is common for this, but under certain conditions (e.g., weak lighting), it may be flawed or unavailable. Bimbo et al. (2015) sought to fill this gap by having a robot identify an object pose by touch. Similarly, Jamisola et al. (2014) have a robot explore a discontinuous surface using only haptic feedback. Focusing more precisely on a held object, Jamali et al. (2014) use force and torque, an end-effector's position, and object behavior in response to rotations to determine how the robot is in contact with an object. We make some use of these ideas in section 4, in the way in which DE NIRO understands whether it has successfully grasped its target object or not, after its arm occludes sight of the object in question.

Recent work on cognitive robotics significantly extends this view. Beetz et al. (2018) introduced a framework that was applied to solve complex manipulation tasks. They stored logical statements in a knowledge base combined with the required data structures for control programs, such as one that makes use of motion planning or inverse kinematics algorithms. In particular, the framework allows an agent to learn generalized knowledge through logging and commonsense knowledge databases. Parts of this framework were already introduced in Tenorth and Beetz (2013).

### 2.3. Work on Learning and Dual-Arm Coordination

Since so many humanoid robots have two arms and are expected to engage with more complex stimuli, task learning with complex, multi-limb movement becomes critical.

Ahmadzadeh et al. (2013) were interested in how efficiently a robot could learn by watching humans. Rather than identifying and tracking objects in the scene independently, this work has a robot recreate the visual relationship among those objects. These researchers expanded their work in subsequent years. As in the research of Rosell et al. (2011) and Ahmadzadeh et al. (2015) used actual human poses as example data to teach a robot by imitation. A human tutor led their robot through the process of learning tasks like pushing, pulling, and categorizing.

Kormushev et al. (2010) show that robots must take advantage of variable stiffness, a technique humans employ by using muscle groups in opposition. They explored a way for robots to self-learn this ability. Our applications rely on passive stiffness and compliance actuators, especially in the form of the arms themselves. Later, these authors explored learning further by exploring which techniques were most effective at teaching humanoid robots more complex tasks (Kormushev et al., 2013). By experimenting with different learning models for many tasks—including pancake flipping, bipedal walking, archery, ironing, whiteboard cleaning, and door opening—they conclude that Reinforcement Learning is most effective for tasks requiring movement in more dimensions or greater flexibility. While our tasks in this paper do not use learning to accomplish their goals, that is a natural area of further exploration, especially given the findings here.

Rakicevic and Kormushev (2019) actually worked on Robot DE NIRO itself. They were interested in how a robot with as many options for movement as DE NIRO might learn a dual-arm task within a defined movement parameter space. They used an active learning approach to teach DE NIRO to shoot an ice hockey puck from a starting position to a goal position. They offer a trial-and-error based task learning system that explores a parameter space efficiently. They also demonstrated how DE NIRO could transfer its learning model to new environments, taking less time to learn a task in subsequent iterations. The dual-arm work is particularly relevant to us, and the task learning model suggests another area to extend our efforts in this paper.

A significant challenge for us in the dual-arm challenge (discussed in section 6) is the points of contact between DE NIRO's arms and the target object. Kanajar et al. (2017), in trying to solve the problem of a bipedal robot climbing over obstacles, found multi-point contact to aid their efforts, rather than hinder them. Jamisola et al. (2016) conducted a deep-dive into dual-arm movement. Their interest was in simplifying the complexity of the dynamics formulation by making dual-arm movement as modular as possible. They treated both arms, in effect, as a single end-effector.

### 2.4. Work on Social Assistance Robots

Social assistance robots for elderly care or general nursing have been subject to extensive research in recent years. They may serve to counterbalance the global nursing shortage caused by both demand factors, such as demographic trends (Tapus et al., 2007), and supply factors, such as unfavorable working environments or egregious wage disparities (Super, 2002; Oulton, 2006). However, most proposed robotic systems aim to directly assist the care recipient—often an independently living elderly person—with social companionship or simple household services (Schroeter et al., 2013; Fischinger et al., 2016). Elderly care today is still predominantly administered by human caregivers who may themselves benefit from a robot assistant.

Robots in aid of the elderly often aim to bolster personal independence with companionship, general household help, and telepresence (Fischinger et al., 2016). These robots are also used for care recipients who suffer from psychological diseases, have disabilities, experience certain illnesses, or are otherwise physically constrained. There has been such growth in these robots that some researchers have surveyed the variety of technologies (Rashidi and Mihailidis, 2013). Examples include:

- Care-O-Bot 3 to support those with limited mobility by Graf et al. (2009),
- ARTOS for emergency support to the elderly by Berns and Mehdi (2010)
- ASIMO by Sakagami et al. (2002),
- HRP-3 by Kaneko et al. (2008),
- DOMEO RobuMate for ambient assisted living by Centre for Applied Assistive Technologies (2011), and
- CompanionAble for social assistance to those with mild cognitive impairments by Schroeter et al. (2013).

Some have built multi-use platforms. These are similar to DE NIRO, which can also perform varied tasks. Willow Garage, for example, built the more general platform PR2, which has been used by various universities to build human-centered applications that include supporting the elderly (Cousins, 2010). Care-O-Bot 3, listed above, also serves many purposes, including cleaning, fetching, and communication (Hans et al., 2002). Berns and Mehdi (2010) built ARTOS, also listed above, to support the elderly during emergencies, and even to “relieve the caregivers from extensive supervision all the time.” Advances for better social interaction and improved navigation have broadened the uses of such robots. Gesture recognition using the Kinect, for example, allows Zhao et al. (2014) to build robots that can receive orders from humans. Gestures are one way DE NIRO could take input from users.

With these recent advances in social assistant robots, public and academic debate has not yet settled on whether such systems are an ethical and desirable outcome for our society (Sparrow and Sparrow, 2006; Wallach and Allen, 2008). For instance, Sharkey and Sharkey (2012) point out six main ethical concerns with robot assistance for elderly care, including human isolation, loss of control and personal liberty, and deception and infantilization. Furthermore, Zsiga et al. (2013) note varying attitudes and preferences regarding social assistance robots, especially across age groups. While this discussion is not settled, we believe it is appropriate in the interim to focus robot assistance on the caregiver. This allows the caregiver to delegate simple, repetitive tasks to a social robot assistant and gain time for more complex, empathetic tasks.

Much less work has sought to support caregivers as they shoulder typical nursing tasks to care for elderly persons. Among those, Ding et al. (2017) propose a transfer assistant robot to lift a patient from a bed to a wheelchair through a model-based holding posture estimation and a model-free generation of the lifting motion. ARTOS, as previously mentioned, frees caregivers from supervision, but not from conducting manipulation tasks (Berns and Mehdi, 2010). Srinivasa et al. (2010) demonstrate complex manipulation skills for bottles and cans based on sparse 3D models for object recognition and pose estimation. They integrate these skills with navigation and mapping capabilities, both in a static and a dynamic environment.

Most of the work we have discussed in this section involves the development of novel robotic platforms of different forms and types. Our work is no different—DE NIRO is also a bespoke platform of hardware and software. Because the robots being developed across this research are not standardized, the research community often has to develop parallel solutions to problems that have already been solved on other hardware platforms. The pick-and-place tasks we perform are not unique to DE NIRO. Nevertheless, DE NIRO's form and sensor suite are unique. Moreover, our aim is to contribute by showing how a generically capable robot can be designed and built rapidly. While social assistance could be an ideal use case for our work, it is not the only application. The ability for a robot to understand a request from a user and retrieve an object of unknown shape and pose from the environment can be applicable more widely.

## 3. Design

In this section, we discuss the design and integration of DE NIRO across its custom grippers, custom exoskeleton, sensor suite, and custom simulation environment.

### 3.1. Core Body

#### 3.1.1. Baxter

The Baxter arms were originally built by manufacturer Rethink Robotics whose IP is now owned by the HAHN Group (Rethink Robotics, 2018). The arms can manipulate objects dexterously. Baxter is designed for collaborative, natural interaction with human users. Each arm has seven degrees of freedom, mainly made of twist and bend joints, allowing pitching and rolling in the shoulders, the elbows, and the upper wrist. The lower wrist, near the hand, can also roll freely. All this affords DE NIRO complex manipulation behavior that one can restrict down to certain zones or axes or enhance by making use of both arms simultaneously (Rethink Robotics, 2015a).

The arms end in mountings with exchangeable grippers, for which we designed two custom grippers (details in section 3.2). Embedded directly in the hand are RGB cameras, which are especially useful when the arms occlude the vision of head-mounted cameras or body-mounted LIDARs. The arms have infrared sensors, accelerometers, and hidden user input buttons in the cuffs. A particular safety feature of the arms is their passive compliance through series elastic actuators (Pratt and Williamson, 1995). This means humans can work in close proximity to the robot safely: in case of contact, the actuators absorb most of the physical impact.

The Baxter screen—positioned where a face might be—is a 1,024 × 600 SVGA LCD that can display custom data (Rethink Robotics, 2015b). Because our goal was to build direct interactivity with humans, the screen was a useful place to give immediate feedback to the user, either by displaying logs (for engineers) or simple, stylized facial expressions (for general users).

#### 3.1.2. Quickie

The Baxter arms are mounted on a QUICKIE movable electric wheelchair base, manufactured by Sunrise Medical (Sunrise Medical, 2019). Its differential drive is operated with a custom PID angular position and velocity controller, allowing primitive motion commands of speed (discrete value between −100 and 100) and direction. The latter is achieved through a PID controller linked to an Inertia Measurement Unit that provides absolute spatial orientation. The navigation controller itself is implemented through an integrated Mbed microcontroller (Arm Limited, 2019). On the hardware side, multiple interleaved safety layers protect users during emergencies. An automated interrupt procedure stops the movement of QUICKIE during timeouts (signal interrupts > 20ms). Furthermore, both on-board and wireless e-stop buttons allow the user to brake the robot immediately.

### 3.2. Custom Grippers

**Figure 2:**
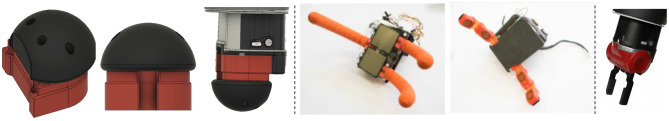
The two custom-gripper designs (left and middle) and the standard Baxter electric gripper (rightmost, Rethink Robotics, 2019).

To conduct manipulation work with Robot DE NIRO, we use three types of grippers: two custom-gripper designs and the standard Baxter electric parallel gripper (Rethink Robotics, 2019), all three illustrated in Figure 2.

Performing heavy, dual-arm object manipulation requires the robot to squeeze large rigid objects. That in turn requires appropriate end-effectors. These are usually non-deformable stumps instead of the commonly found actuated “fingers.” We first tried the simplest design of a solid rectangular block, though this required the arms to always be orthogonal to the object being manipulated. Small errors in pose estimation of the target could lead to it being pierced by the edge of the block. To avoid this, we opted for a spherical end-effector, which adds an additional degree of freedom around the contact point. This comes at the cost of more robust disturbance adaptation, as the spherical shape reduces the overall contact area.

The second custom-gripper is a bio-inspired design with soft fingers that can bend. It is available in two different design variants—soft joints made of rubber and a flexible skeleton structure. In both variants, a dynamixel motor pulls a tendon to close the fingers simultaneously in a claw-like behavior, allowing it to grasp larger objects (in comparison to the standard Baxter end-effector) that may require a wider span. Furthermore, the bio-inspired gripper is equipped with force sensors that can achieve haptic feedback. The bio-inspired gripper is accessible via SSH protocol (Hentsch, 2017).

### 3.3. DE VITO—Custom Exoskeleton

**Figure 3:**
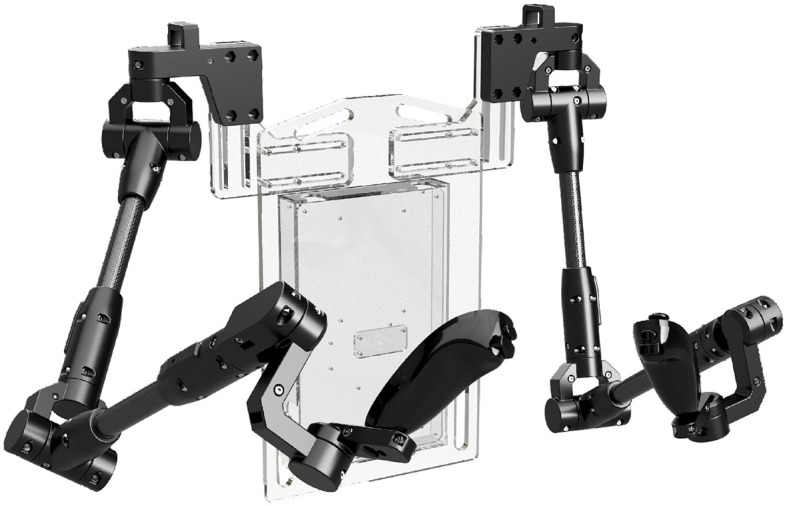
CAD model of the DE VITO, a dual-arm, upper-limb exoskeleton for semi-autonomous robot manipulation. Source: Falck et al. (2019).

DE NIRO is designed as a research platform for autonomous operation. In spite of the fast progress of manipulation research, DE NIRO might be faced with unfamiliar situations. In such scenarios, semi-autonomous control through a remote human user could be helpful. For this reason, we built a custom passive upper-limb exoskeleton named DE VITO (Design Engineering's Virtual Interface for Tele-Operation), described in more detail in Falck et al. (2019) and depicted in Figure 3. DE VITO allows dual-arm control with seven degrees of freedom. It is lightweight, inexpensive, and fully open source (Falck et al., 2019).

### 3.4. Sensors and Hardware Augmentation

DE NIRO also has the following sensors and other hardware:

- A Microsoft Kinect RGB-D camera (placed on top of DE NIRO with a rotating, controllable mount)
- Stereovision cameras with built-in microphones
- A 2D LIDAR scanner (Hokuyo UTM-20LX)
- A 3D LIDAR scanner (Hokuyo UTM-10LX mounted on a servo motor (Dynamixel MX-28T) with slip ring)
- A custom 360-degree camera rig of six Logitech c920 HD webcams (assorted equidistantly)
- One Linux-based laptop to run our primary software
- One Windows laptop to receive navigation data from the Kinect SDK
- One local area network router to link the various computing systems, including the Baxter core
- An Amazon Echo Dot device for audio input
- Logitech speakers for audio output.

Note that both the 3D LIDAR scanner and the Kinect are capable of producing 3D point clouds. All of these actuators and sensors together make up Robot DE NIRO. When we refer to DE NIRO, we are generally referring to all these features and their resultant capabilities.

### 3.5. ROS Usage

We used the open source Robot Operating System (ROS) as middleware. ROS offers design transparency and an active community of robotics developers. Its core value to us is in allowing us to link together all the sensors and actuators described in section 3.4. The central challenge of a robot built from independent component parts, like DE NIRO, is communication across the components. If the Kinect identifies a target object position, that information needs to be passed to the Baxter arms, and the arms have to know where they exist in precise relation to the Kinect. That connective information is what creates a robot out of these disparate parts attached to one another.

**Figure 4:**
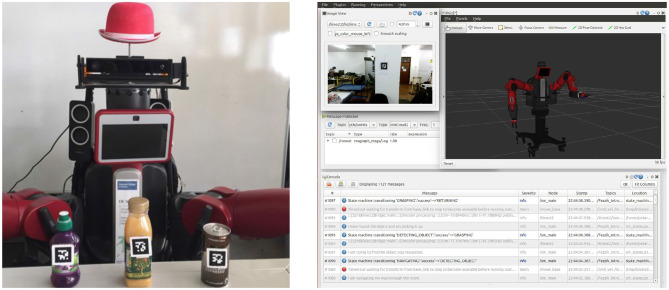
The 2D fiducial markers attached to test objects **(left)** and the RQT-based technical GUI for testing purposes **(right)**.

All these components were linked programmatically by ROS and literally by the local area network router. In addition, to test, debug, and integrate log outputs, we built an RQT-based GUI illustrated in Figure 4 (right), mainly of use to the technical user (Thomas et al., 2016).

While ROS is prevalent in the industry, there are many ways to use it, and two ROS-enabled projects may have very different characteristics. ROS is a very thin software framework by design (Open Source Robotics Foundation, 2019), and it works best by allowing communication across small, independent collections of code. The roscore command forms the basis of ROS's decentralized architecture and knits together all of ROS's nodes (Quigley et al., 2015). It initiates a name service so each new node can register itself, look up other nodes' addresses (the ROS master), and access a globally available data store (the parameter server). All other logic lives in independent code modules and on ROS's federated node graph.

As Quigley et al. (2015) notes, ROS provides various methods of communication, each with pros and cons. Our challenge is in selecting the correct methods for the correct tasks. In our case, starting up the Baxter base runs roscore, so we could at least register all independent subcomponents to that same ROS master. The simplest communication method are ROS publishers and subscribers that regularly post to or read from ROS nodes. This was most useful for continuous, low-level data transfer. Our navigation module, for example, published odometry data continuously. ROS services are also available, which can send off parameters to a function elsewhere which executes and can send back a return value. These are best during specific call-and-response moments. This is in fact how we passed the target object location from the object recognition module to the grasping module. There are also ROS actions, to communicate data across a duration of time, particularly useful for extended processes, such as movement. Accordingly, we used this to track the robot's position when moving to the object warehouse. Finally, there is the parameter server, which holds a globally accessible dictionary and can be used when performance is not a concern. We used this for DE NIRO to identify and save the active requesting user at the beginning of the fetch routine. We discuss these all further in section 4.

We broke the tasks of this fetch routine into various independent subsystems, such as navigation, grasping, audio input, and so on. Each subsystem takes input, processes it, and produces output differently. Within the object recognition subsystem, for example, DE NIRO receives an instruction from the speech recognition subsystem and uses that indicator to seek out a match in real-time.

### 3.6. Gazebo Simulation

To safely test manipulation tasks before deploying them on the physical robot, we built a simulation of DE NIRO using Gazebo, which runs the Open Dynamics Engine (ODE) physics engine (Koenig and Howard, 2004). Gazebo is used specifically for robot simulations with an emphasis on accurate friction and torque models, especially as compared to other popular engines like Mujoco. The virtual representation of DE NIRO's model is described in the Unified Robot Description Format (URDF). URDF specifies the position of joints and linkages and requires highly-accurate intrinsic parameters, like mass, inertia, collision topology, and surface friction. Some (like mass) must be exact, while others (like friction coefficients), can be greatly exaggerated to improve contact with the simulated objects.

**Figure 5:**
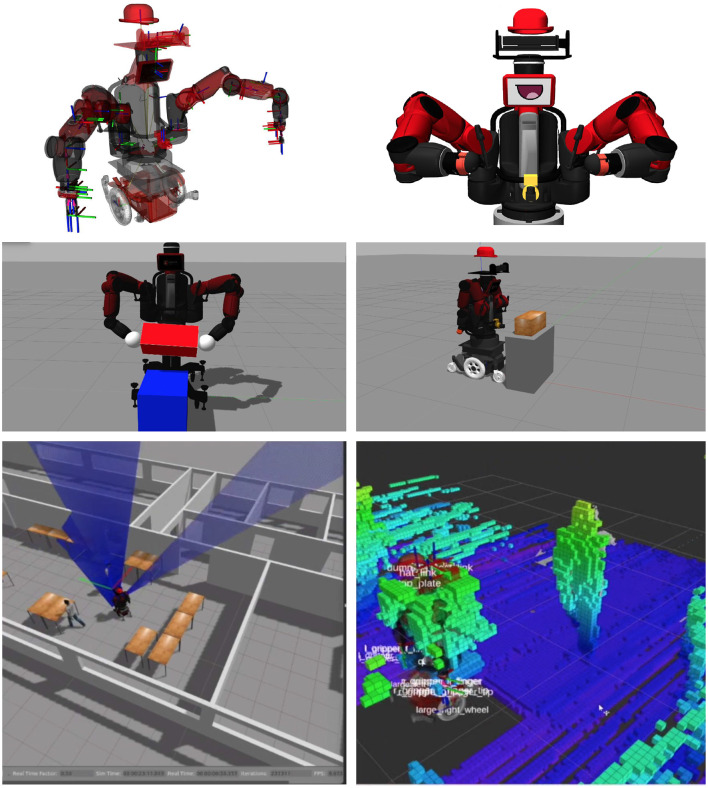
Simulation model of DE NIRO in Gazebo. **Top**: The simulation model of DE NIRO with visible link transformations, rendered in RViz **(left)** and Gazebo **(right)**. **Middle**: Two manipulation scenarios with the default Baxter description **(left)** and the complete simulation model of DE NIRO **(right)**. **Bottom**: Example of the robot being aware of a human obstacle using the simulated sensors yielding 3D point clouds.

In addition to simulating DE NIRO's Baxter upper-body, Gazebo also provides a useful plugin system to recreate the sensor architecture, including the two LIDAR scanners, the Kinect, and the QUICKIE wheelchair differential drive. This complete simulation model of DE NIRO is stored in a YAML file description and is particularly important for collision avoidance in the implementation sections discussed below. We show the complete simulation model of DE NIRO in various manipulation scenarios in Figure 5.

## 4. Implementation: Fetch Routine

In this and the following two sections, we present three generic skill challenges and describe how we implemented solutions to them:

1. Fetch Routine
2. Automatic Object Detection
3. Dual-Armed Grasping.

### 4.1. Overview

In many domestic support roles, an assistant robot may be required to fetch specific objects from certain locations in the home. Our first goal was to have DE NIRO interact with non-specialists to fetch objects autonomously.

Our fetch routine consists of a series of modular components that are each called by a finite-state machine in a particular order. A successful flow of the robot through a fetch task involves these activities:

- **Speech Input:** DE NIRO receives a verbal command from an approved human user
- **Audio Output:** It regularly confirms its actions through vocalizations
- **Navigation, Mapping, and Trajectory Planning:** It moves to an object warehouse
- **Object Recognition:** It identifies the right object with the help of 2D fiducial markers
- **Grasping:** It grasps that object
- **Returning:** It returns to the point of origin
- **Face Recognition:** It only releases the object to the approved human user.

### 4.2. State Machine

The state machine determines the right points at which to activate DE NIRO's actuators, sensors, and computers. The state machine architecture supports significant complexity, though ours is relatively linear, with DE NIRO returning to idle after completing (or erroring out of) the above process. The state machine greatly simplified our design—with it, we only needed to consider the components and programs required for a given action.

**Figure 6:**
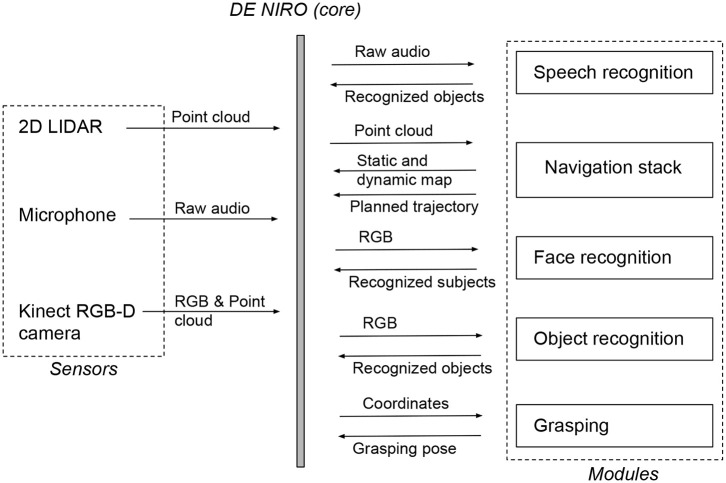
Flow chart of the main software modules introduced in section 4 which are integrated and connected through ROS, also illustrating data inputs and outputs.

To build the state machine, we used the SMACH package (Bohren, 2018). This allows each state to be built with the same general structure, linked closely to the rest of the ROS framework. From within a SMACH state, we could make calls to the ROS parameter server, we could activate a particular ROS module, and we could send data from one component to another upon entry to or exit from a state. An overview of the modular software architecture in section 4 is illustrated as a flow chart in Figure 6.

### 4.3. Speech Input

There are many ways for DE NIRO to identify the target object. Raw typed input into the terminal works and could be generated by a simple mobile application with pregenerated commands. Visual input also works by showing the robot a marker it could match to a target. We used both of these methods during development. However, for non-specialists, voice commands seemed simplest and most natural. We needed a way to accurately detect and classify relevant voice commands, despite variation across human voices, ambient environments, and network strengths.

We tested a variety of audio recognition methods:

- The Amazon Web Services (AWS) Alexa speech-to-text service (Amazon Webservices, 2019)
- The Google Cloud speech-to-text conversion technology (Google Cloud, 2019a)
- The CMUSphinx Open Source Speech Recognition Toolkit (Carnegie Mellon University, 2018b)

We tested AWS using Amazon's Echo dot hardware to listen for particular command structures. The Echo sends an audio file to be converted, stringified, and returned by AWS. Unfortunately, the network connection to AWS proved problematic. We experienced frequent connection failures to AWS. Practically, AWS's requirement of a specially configured web socket, to be approved by an IT administrator, slowed down our process as well. AWS needs all this because it purports to translate almost any audio into almost any text, including custom words (Hunt, 2018). Google's solution interfaced more seamlessly with our software. It too offered to interpret almost any words spoken by users. Over multiple tests, it did so well. However, it was also very sensitive to ambient noise. Audio input had to be spoken directly into a microphone, or else audio would have to be preprocessed before sending the file to Google.

Both AWS and Google relied on neural networks to make their speech to text conversions broadly applicable. However, we did not need such wide usage, especially when considering the specific support role the robot would be in. Our design challenge was to retrieve requested objects in a dynamic environment, perhaps in aid of a human caregiver. More importantly, requiring an active connection to the internet could be a challenge. If it were possible to do speech transcription locally, that would be preferable.

The third solution, CMUSphinx, was an open source package that did not make use of a neural network, but rather relied on more conventional methods, trading off breadth for simplicity. It is explicitly designed to support “low-resource platforms” (Carnegie Mellon University, 2018a). While CMUSphinx was also vulnerable to background noise, it had a library to improve accuracy by masking ambient sounds. Best of all, it worked without an internet connection. To simplify and speed up CMUSphinx, we applied the JSpeech Grammar Format (JSGF) to CMUSphinx. This format allows CMUSphinx to reduce the space of possible audio input by providing an expected grammar, in which certain keywords are anticipated in a particular order. For example, we set our grammar like this: “[DE NIRO, please] [fetch, give, get] [me] a[n] [object].” JSGF allowed this combination of an explicit format that could be taught to users with specific verb and object selections and optional elements (like the word “please”). With the speed gains from JSGF, we could offer users a selection of commands that, while not exhaustive, would cover the range of likely sentence formats, and to which DE NIRO could respond with dispatch.

### 4.4. Audio Output

Audio output helps DE NIRO communicate to its human interlocutors. It was a surprisingly useful technique to give key status updates asynchronously to the user while DE NIRO was engaged in other tasks. In addition, we observed anecdotally that users appeared much more interested in engaging with DE NIRO when the robot seemed to talk directly to them.

We made use of the eSpeak text-to-speech synthesizer (eSpeak, 2018). eSpeak can be sent any string in English and then produce appropriate vocalizations through a computer's speakers. eSpeak does not rely on actual human voice recordings, but instead produces a formulated mechanized voice. For DE NIRO, a robotic voice actually seemed to be an engaging feature rather than a problem. Like CMUSphinx, eSpeak was open source and worked without an internet connection. Here again, we explored usage of equivalent text-to-speech sources linked to Amazon's (Amazon Webservices, 2018) and Google's (Google Cloud, 2019b) cloud services, but the requirement for a direct internet connection was the deciding difference.

Because eSpeak is quite small and does not need to sort through a library of audio waveforms, it exhibited little lag between an instruction being sent and the vocalization being heard. Each individual text to speech instruction is sequenced and outputted in a row before the next one began, avoiding problems with overlapping audio output. Unlike with other components of this fetch routine with more specific focuses, like grasping, DE NIRO used audio output in each and every state.

### 4.5. Face Recognition

Recognizing an individual human face is a necessity in a domestic environment. Manipulation intelligence requires more than just an adjustment to actuators. It requires physical movement and change in response to meaningfully relevant information. In the domestic environment, it might be a matter of significant medical importance that the robot gives the right payloads to the right people. The robot must not give dangerous medicine to a child, for example. We anticipated a busy home environment, with perhaps multiple moving faces present in the camera frame. We needed to reliably distinguish the foregrounded face and compare it against all the approved users.

For facial recognition, we used a straightforward, but robust, machine learning model. We uploaded facial photos of approved users into a facial recognition ROS module. After DE NIRO receives its verbal command, the module then takes live image frames from the Kinect camera (Geitgey, 2018). It runs these frames through a pre-trained model built from a Residual Network (ResNet) (He et al., 2016). This model has reached a 99.38% accuracy on a standard benchmark (King, 2018). It compares vector encodings of known reference faces with those extracted from the processed frames by computing a distance metric. It predicts positively (i.e., a matching face) if this distance is below a threshold. We tuned the model to predict with a very low false-positive rate at the cost of a slightly increased false-negative rate, in order to be less vulnerable to unintended interactions, but potentially requiring several frames as input (at 30 Hz) before a positive interaction would be predicted (Microsoft, 2014).

In practice, we never experienced any problems in recognition accuracy. Even when lighting was relatively poor, like in our laboratory, or when conditions were visually crowded, like when we demonstrated DE NIRO to a public forum full of jostling families, DE NIRO was always able to accurately separate one face from another.

### 4.6. Navigation, Mapping, and Trajectory Planning

Moving to and from the object warehouse is perhaps the most vulnerable part of the fetch routine. The robot needs to know where it is located in a 2D space, react to unexpected dynamic obstacles that move into its way, send a message to power the wheels sufficiently, recalibrate and redirect if the physics of the space fail to result in the movement anticipated (if the robot is on a high friction surface like a carpet, for example), and iterate this process regularly until arriving at a destination. Our goal here was to identify and incorporate a reliable, robust navigation stack. We did not attempt to contribute a novel technique for robotic movement—just one that worked for DE NIRO. Because we used these features when DE NIRO went to and from the object warehouse, our goal was to solve a straightforward there-and-back scenario.

#### 4.6.1. Navigation

To power navigation, we used Simultaneous Localization And Mapping (SLAM). SLAM seeks to help a robot map a particular space and identify its pose in that space at the same time. Our use case, like most that utilize SLAM, involves a robot in motion, making self-positioning a particular challenge. SLAM's central challenge is that the robot must first determine its pose and each possible landmark, and then update that understanding upon each new observation. That leads to an exponentially rising problem space (Durrant-Whyte and Bailey, 2006).

The complexity here comes from the dynamism and uncertainty of the environment. However, our expected environment was not highly dynamic in terms of its fixed features—walls and tables were not likely to move quickly or frequently. People might move or walk around, and so collision detection would be important. Nevertheless, fixed features could simplify this challenge by giving DE NIRO more spatial information before it needed to operate freely.

To do this, we moved DE NIRO through its environment ahead of time with the scanner active. It generated a bitmap which it updated and saved locally with each new traversal. We did this using the full robot, but there is nothing that would prevent a local, handheld scanner from accomplishing the same task. As long as it built a map that was accurate to the pose of the robot, it could be sent separately and used by DE NIRO for navigation. All this provides DE NIRO the advantage of operating in a situation with a known ground truth.

#### 4.6.2. Localization

We simplified our problem space further by adding another constraint to our use case: DE NIRO would only use a 2D map with no major level changes. DE NIRO therefore navigates within a 2D bitmap and only needs to calculate pose estimates in a 2D space.

To further improve navigation and localization accuracy, we made use of the very high update rate of the 2D LIDAR scanner, which DE NIRO used to generate its map and to move within that map. ROS has a variety of possible SLAM packages to use, but given our 2D constraint, we selected the hector_slam package, built by Kohlbrecher et al. (2011). This package does not need to rely on odometry to determine pose. Hector mapping can derive odometry on the basis of wheel velocity, but it is not required. Our robot uses a QUICKIE wheelchair base linked to a customized Mbed microcontroller. Even if we could deduce odometry on the basis of wheel velocity, the accuracy would likely be low. The higher quality Hokuyo LIDAR scanner was a more reliable way to estimate the robot's pose.

**Figure 7:**
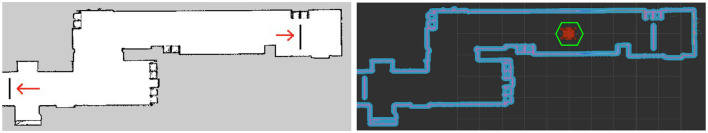
**Left**: A static map of a corridor. The red arrow points at a synthetic barrier manually added to the map. **Right**: A costmap of 10 cm in blue surrounds all static barriers. The green hexagonal shape is a synthetic barrier around the QUICKIE base used for collision avoidance.

Using hector_slam, the robot localizes itself by applying a dynamic map as an additional layer over the static 2D bitmap (Kohlbrecher, 2018). This dynamic map adjusts as the robot moves through the space it already knows. To add an extra safety barrier around any static and dynamic obstacles in the way, we applied a costmap around all artifacts. Because DE NIRO is wider at the top than at the bottom, and because the wheelchair base is handling movement and navigation, we needed extra buffer to be sure that DE NIRO gave any obstacles a wide enough berth. We padded objects by 10 cm. As an additional safety cushion, we added a synthetic hexagonal barrier around the QUICKIE base. We chose a hexagon instead of a direct vertical projection of the robot for simplicity. DE NIRO has multiple computers and sensors strapped to itself that are frequently being removed and reattached. For DE NIRO to know its exact proportions at all times, it would need further sensors just to monitor its own shape and any new protrusions. The conservative constraint of a hexagon allowed DE NIRO to robustly plan trajectories, get out of tight spaces, and efficiently avoid collisions. The static map together with its costmap is illustrated in Figure 7.

#### 4.6.3. Trajectory Planning

**Figure 8:**
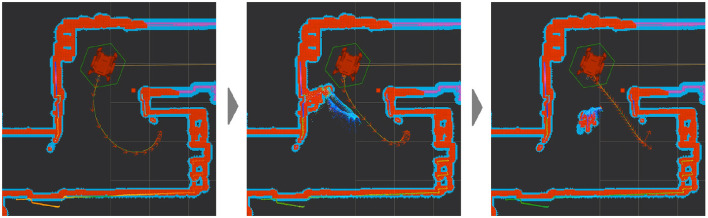
The original optimal path **(left)**, encountering a dynamic obstacle **(middle)**, and adjusting in response **(right)** during trajectory planning.

DE NIRO crafts the trajectories themselves with a “timed elastic band” approach (Rosmann et al., 2013). Each trajectory from initial pose to end goal, is an optimization problem with various stages. The goal is to reduce total travel time to a destination and proximity to any obstructive barriers along the way (Rosmann, 2018). With this work, DE NIRO can update its existing map, quickly identify new obstacles, and plan paths around such items. The planned velocities manifest as spurts of power to the wheels. This works through the use of our custom Mbed microcontroller, which scales the velocities appropriately and reduces their variance, such that each one can be linked continuously. From there, the Mbed converts those velocities into an electric signal that it sends to the motor, which finally drives rotational movement in the wheels (Aveiga, 2017; Arm Limited, 2019). The minuteness of those velocities allows the robot to navigate around new obstacles, given enough time and space. If a human stands in the way of the robot's most efficient path, and if the robot sees it ahead of time, it can plan an alternate trajectory around it. The dynamic trajectory planning in a scenario where an obstacle (human) blocks the way is illustrated in Figure 8. If DE NIRO lacks enough time to plan an alternate trajectory or if an encroaching object is within its collision zone, it will stop moving until the object is cleared and it can plan a clear path again.

### 4.7. Object Recognition

By this stage, DE NIRO has finally arrived at the object warehouse. In a domestic environment, this could be a medicine shelf or a pantry closet. We used a desk or shelving unit at different heights and locations around the navigable space. We placed a collection of objects on this shelf. After experiencing occasional failures in grasping, we typically arranged three possible objects (usually various shapes of bottles) in a horizontal line, with some space between them all. The challenge for the robot was to identify the correct object and its coordinates in 3D space.

We found an excellent solution in 2D fiducial markers, derived from the chilitags library (Lemaignan, 2018). The 2D fiducial marker, like a barcode, could be printed directly on bottle labels or printed onto stickers that could then be attached to individual objects. Once the fiducial markers were in the camera frame, DE NIRO could quickly, effectively, and precisely recognize them and their coordinates, even with poor lighting conditions, unusual angles, or lengthy distances. Very rarely, the coordinates identified for an individual image frame of the fiducial markers might be volatile. Reflections or matte surfaces might lead to highly inaccurate coordinates on a very infrequent basis. To solve for this, we recorded an array of 20 frames (inputted in less than a second by the Kinect camera), and selected the median of the poses estimated from each frame.

We did explore other more generic and scalable solutions. However, for this manipulation challenge, none of our methods matched the reliability of the fiducial markers. For example, we explored a machine learning algorithm to allow DE NIRO to identify any translucent bottle before it. But this requires the robot to learn about the objects it might be expected to grasp ahead of time. Domestic users actually have greater flexibility in object types with a simple solution like stickers. Nevertheless, identifying the pose of an object is such a core requirement for manipulation intelligence that we do devote more time to this challenge, and we detail this later in section 5.

### 4.8. Grasping

#### 4.8.1. Arm Movement

After the Kinect identifies the relevant fiducial marker, it locates it in its 3D viewspace. To actually grasp those objects, the pose must be converted to a coordinate frame that the arms can interpret. DE NIRO's Kinect sits above its central screen, horizontally aligned with the base of the Baxter arms. As a result, it is necessary to know the precise position of the Kinect relative to the Baxter base frame in order to apply the appropriate transform. This is particularly difficult because of the rotatable camera angle of the Kinect camera. We discuss the calibration process more in section 5.2.1.

To control all seven joints in the arm, we made use of an inverse kinematics solver that could, based on a target end-position, calculate the necessary sequential joint angles, and therefore the arm trajectories (Rethink Robotics, 2015c). When taking this action, DE NIRO was acting blind to other objects in the environment, such as the other Baxter arm. DE NIRO could accidentally collide with an overhanging shelf or even another part of its own hardware. To try prevent collisions near the point of grasping, we sent DE NIRO an intermediate target position about 10 cm between the fiducial marker and itself.

**Figure 9:**
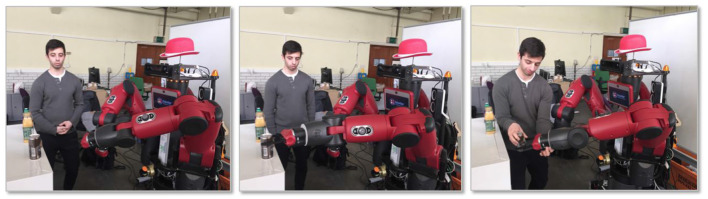
The three grasping stages of moving to an intermediate position **(left)**, grasping the object **(middle)**, and executing handover-mode **(right)**. Written consent for using this figure by the person depicted has been obtained.

DE NIRO would first reach there with its two-finger pincers separated widely enough for any bottle. After pausing briefly in that intermediate position, DE NIRO moves forward that final 10 centimeters to the true object position. DE NIRO only knows the location of the marker; not the width or depth of the object the marker is attached to. So it moves the hand forward until the base of its “palm” is at the original marker coordinates. Then, it pinches together its two fingers until encountering slight pressure. This indicates that it has grasped the object. This procedure worked in practice surprisingly well as a initial solution, and we illustrate it in Figure 9. However, in later challenges, we presented other methods for DE NIRO to more inherently understand object geometry and apply more complex manipulation behavior.

#### 4.8.2. Adaptation

In the Grasping state, we added extra logic to have DE NIRO react more naturally when obstacles arise during the grasping process. At least at this stage, the adaptation was more about error handling than true autonomy. For example, if DE NIRO is unable to reach the object (perhaps the object is behind a transparent barrier), it would pause and revert its hand to where it was before. Then, it would try again, just in case its arm movement decision had been ineffective. DE NIRO's grasping component reports each failure up to the state machine, which directs DE NIRO to give up after the fifth failed attempt and proceed to the next state, Returning.

In addition, based on what DE NIRO perceives, it will select which arm to use autonomously. If the target object in the object warehouse is to the left of DE NIRO's central axis, it will begin the grasping attempt with the left arm, and if to the right, then the right arm. Moreover, if the target object moves (say, from one side to another) after DE NIRO begins its arm movement, DE NIRO will pause and try again with the opposite hand. If the stationary arm is in the way of the moving arm, DE NIRO will dynamically move the blocking arm out of the way to avoid collisions.

### 4.9. Returning

After obtaining the object, DE NIRO, which stands nearly two meters tall, holds its payload with its arm raised, out of reach of any children or other disturbances it may encounter on its way back to its origin. It returns back to the point of origin and begins seeking out the original requester, using the same method for facial recognition it applied before. If it sees someone other than the original user, DE NIRO will simply say so with audio output, indicating that it requires the original user to release its object. Only when the correct user is before it will DE NIRO lower its arm and release the final payload. To do this, the user needs apply a small force on the hand along the z-axis. By pulling on the payload upward or downward, the robot will release its grip, providing the user with the object.

### 4.10. Discussion

Our work in response to this challenge was a measured success. By the end, DE NIRO could receive a command, navigate through a room, fetch an object, and return it to the requesting user. However, there are some significant areas of future research to explore before DE NIRO could be converted into any sort of commonplace household robot.

There is also more nuance that can be applied to this process. DE NIRO does adapt fluidly during the grasping process, switching arms or trying multiple times when a grasp fails. However, DE NIRO is generally focused on one task at a time—it does not take in data from all its sensors at all times. An alternate solution might have DE NIRO even more aware of its environment while moving or while grasping, so that it can adapt to a number of potential other objects. A more complex state machine might also be a simple way of implementing far more complex looping and failure behavior. At the moment, DE NIRO selects between two locations—a point of origin and an object warehouse. It would not require much more work to offer a variety of object warehouses and indicate them by audio command, such that one could ask DE NIRO to fetch a book from an office room, but a bottle of water from the kitchen.

This challenge was our first step into exploring how Robot DE NIRO would work in a real environment, in which its ability to manipulate objects was central to its success. Next, we concentrate on the challenge of manipulation and grasping to see where and how we can widen DE NIRO's set of actions.

## 5. Implementation: Motion Planning for Single-arm Object Manipulation

### 5.1. Overview

After our work on the fetch routine, there were a number of promising areas of future research. What stood out to us was how DE NIRO could behave more intelligently once at the object warehouse, when identifying its target and moving to grasp it. Our next research goal hoped to have the robot better understand and act upon its grasping environment. There were three sub-challenges in accomplishing this goal:

1. **Vision:** DE NIRO needed to better understand the surface upon which the objects stood, the ways in which those objects were arranged, and the primitive shape of the objects themselves.
2. **Motion Planning:** During our fetch routine, the arm motion planning sometimes encountered errors, and we needed a more robust process to plan arm and hand movement.
3. **Grasping:** DE NIRO needed to identify and execute the hand pose necessary to successfully take hold of an object.

Our fetch routine required a very particular placement of objects, in a very precise order. For this challenge, we aimed to have DE NIRO conduct its behavior in an unknown object warehouse, where the robot is not expecting one singular type of object or position. We felt that in a true domestic environment, cognitively-capable robots would distinguish themselves by adaptation to their environment.

### 5.2. Vision

Our first aim was to estimate an object's primitive shape, to segment one object from another, and detect an object's pose. We restricted DE NIRO's search to a fixed set of object geometries directly on the planar surface. It provides a constraint to the human user by limiting the zone of interaction to a clearly defined workspace. That also simplifies point cloud processing during the vision pipeline. We use a custom C++ script that draws inspiration from the source code of the ROS tabletop_object_detector package (Willow Garage, 2018) and makes use of the Point Cloud Library discussed in detail below (Rusu and Cousins, 2011). While both the Kinect and the 3D LIDAR can produce 3D point clouds, we used the Kinect for this purpose. As in our fetch routine, the fact that the Kinect was mounted at the top of DE NIRO and could be angled up and down provided the highest granularity for objects in front of DE NIRO.

Since we wished for DE NIRO to be able to perceive its objects in an unknown environment, we had to design perception software that could account for unpredictability. As a design principle, we had to plan for unstable perception: objects might be arranged in any way, and might be overlapped from the perspective of the Kinect camera. Our software needed to account for this.

#### 5.2.1. Preprocessing and Calibration

To preprocess the raw 3D point cloud, we downsample it into a voxel grid that only returns the points located at less than 1.5m in the positive z direction of the Kinect—this method eliminates potential background points from the floor, which also represents a planar surface. Then, we apply a Gaussian filter to eliminate outliers or aberrations.

**Figure 10:**
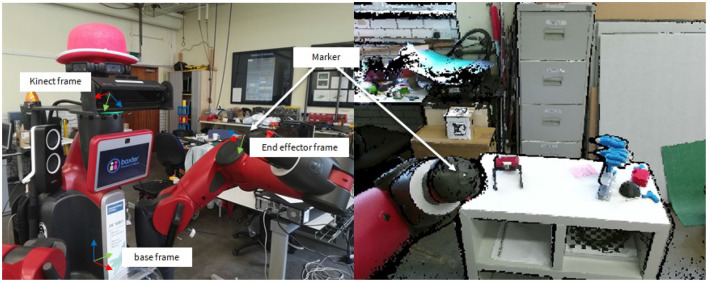
Protocol for calibrating the Kinect depth camera relative to the Baxter base frame. **Left**: DE NIRO's pose during one random position of the calibration protocol. **Right**: The point cloud as seen from the Kinect depth camera.

As in our fetch routine, this requires calibrating the position of the Kinect. To do so, we first stick a fiducial marker to both manipulation grippers of DE NIRO. Then, while the Kinect camera is turned on, we manually place the grippers at several random positions in DE NIRO's operating area (confirmed by a user input). For each position, we store the position of the marker in the depth camera frame (returned by the Kinect node) and in the base frame (returned by the Baxter arms' core API). Finally, we apply a least squares method to optimize the transformation between the Kinect frame and the base frame. For each camera position, we conduct ten repetitions and validate our results quantitatively. Figure 10 further illustrates the calibration protocol.

#### 5.2.2. Segmentation and Detection

**Figure 11:**
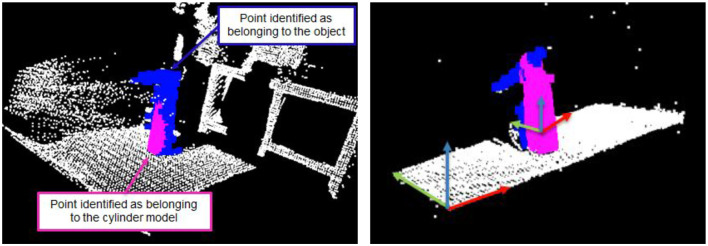
Application of the RANSAC algorithm to segment a cylindrical shaped object after several iterations **(left)** and after convergence **(right)**. Points marked in blue belong to the object being modeled (here: a bottle), and points in purple are recognized by the RANSAC algorithm as belonging to the cylindrical model. Furthermore, the right figure illustrates the first three principal components of the two objects at hand.

Our next task is the meat of this project: to simultaneously detect and segment a fixed set of objects. In our arrangement, these objects included a flat surface, a cuboid, and a cylinder, although any 3D geometric shape could be used. Note, however, that the list of possible object geometries must be known before segmenting the point cloud. We use the RANSAC algorithm, which chooses a random subset of the data and iteratively fits the parameters of a geometric model (e.g., the radius and height of the cylinder) to the given data distribution (Fischler and Bolles, 1981). By looping over a database of candidate models, one can detect which objects match by selecting whichever minimizes the error between the parameterized object model and the empirically observed datapoints. In this challenge, the RANSAC algorithm performed very robustly. After RANSAC terminated, those points lying within the model were classified as belonging to the object. Figure 11 illustrates the RANSAC algorithm applied to a cylindrical object.

#### 5.2.3. Pose Estimation

In the second step, we used the subset of points belonging to the object model in order to determine its pose. That pose is necessary to fully reconstruct the planning scene. We used Principal Component Analysis (PCA), which identifies the directions of largest variability (the principal components) in each model (Wold et al., 1987). For example, in a plane subset, the largest variability is likely to be observed along the length, width, and height of the object. The principal components are subsequently used to compute the rotation of the object. The translational disposition of the center of the object is directly obtained from the position of the geometric model, yielding its full pose. Figure 11 (right) illustrates the first three principal components of both the planar surface and the cylinder model. The parameters and pose of each detected object are communicated across the robot using custom ROS messages.

### 5.3. Motion Planning

Given the understanding of the objects and their poses in the scene, at this point, DE NIRO must determine an optimal trajectory from its initial arm pose to the position of the target object. We used MoveIt! with the moveit_python library as a high-level binding to configure and plan a trajectory across numerous pathplanning algorithms (Sucan and Chitta, 2019). After several initial trials, we decided to use the RRT^*^ algorithm with a constraint on the maximum computation time of 2 seconds in order to ensure real-time feasible responses (Karaman and Frazzoli, 2010). RRT^*^ is a well-established, Rapidly exploring Random Tree (RRT) method supporting dynamic environments and both holonomic and non-holonomic constraints. Furthermore, it guarantees asymptotically optimal properties by introducing tree rewiring and best neighbor search (Noreen et al., 2016). The RRT^*^ algorithm yielded faster, more reliable convergence and better planned paths in comparison to various benchmark algorithms (RRT, RRT Connect, KPIECE, and PRM).

### 5.4. Planning Scene

The planning scene is constructed by first inserting the recognized objects with their given pose and geometric dimensions. Each object is flagged as a potential obstacle, in order to avoid collisions with the planned trajectory. In addition, we specify three target poses during the pick-and-place procedure and compute a path between pairs of them separately: First, a *grasping pose*, corresponding to the pose of the end-effector when grasping an object. The grasping pose is further discussed in section 5.6. Second, an *approach pose*, corresponding to a pose close to, but with a non-colliding offset from the grasping pose and in the same rotation. Splitting the motion into these two target poses allows, as already seen in the fetch routine, a chance to encapsulate the movement and the actual grasping procedure more explicitly and avoids a potential collision detection with the object grasped. Third, a *basked pose*, the goal where the object is placed as soon as it has been picked successfully.

### 5.5. Path Planning Optimization

To obtain a planned trajectory, we formulate the path planning problem as an optimization that jointly minimizes the cost of two terms:

1. The Median Derivation Joint (MDJ) cost *c*_*MDJ*_ measures the Euclidean distance between the joint angles θ_*p*_ of each point *p* ∈ *P* and the average joint angles θ_0_ ($\theta_{0},\theta_{p}\in {\mathbb{R}}^{7},\forall p\in P$) as follows:
  $$\begin{array}{l} c_{MDF}=\underset{p\in P}{\sum }{\left(\theta_{p}-\theta_{0}\right)}^{2} \end{array}$$
  θ_0_ corresponds to the most frequently visited joint angles of the Baxter arm. Intuitively, the *c*_*MDJ*_ cost component penalizes trajectories which approach the joint limits and therefore encourages a simpler motion, also protecting the actuator hardware.
2. As the second cost term, the End Effector Position (EEP) cost *c*_*EEP*_ which measures the Euclidean distance between the trajectory points *x*_*p*_, *p* ∈ *P*, where P is a set of indices of points along a trajectory, and the target point *x*_*T*_ (translation component of target pose) of the end-effector, $x_{p},x_{T}\in {\mathbb{R}}^{3}$, as follows:
  $$\begin{array}{l} c_{EEP}=\underset{p\in P}{\sum }{\left(x_{p}-x_{T}\right)}^{2} \end{array}$$
  Intuitively, the *c*_*EEP*_ cost component penalizes complex, curvy trajectories, favoring more direct movement toward the target.

The path planning optimization problem is formulated as a convex combination of the two cost terms as follows:

$$\begin{array}{l} \underset{\begin{array}{c} \theta_{1},\theta_{2},\ldots ,\theta_{P};\\ x_{1},x_{2},\ldots ,x_{P} \end{array}}{\text{min}}\alpha \cdot c_{MDJ}+\left(1-\alpha \right)\cdot c_{EEP} \end{array}$$

α is a weighting factor that allows us to prioritize the effect of one of the two cost terms. After empiric fine-tuning, we set it to α = 0.005.

### 5.6. Discussion

In this section, we qualitatively and quantitatively assess our second challenge of manipulation attempts with DE NIRO.

#### 5.6.1. Grasping Pose

**Figure 12:**
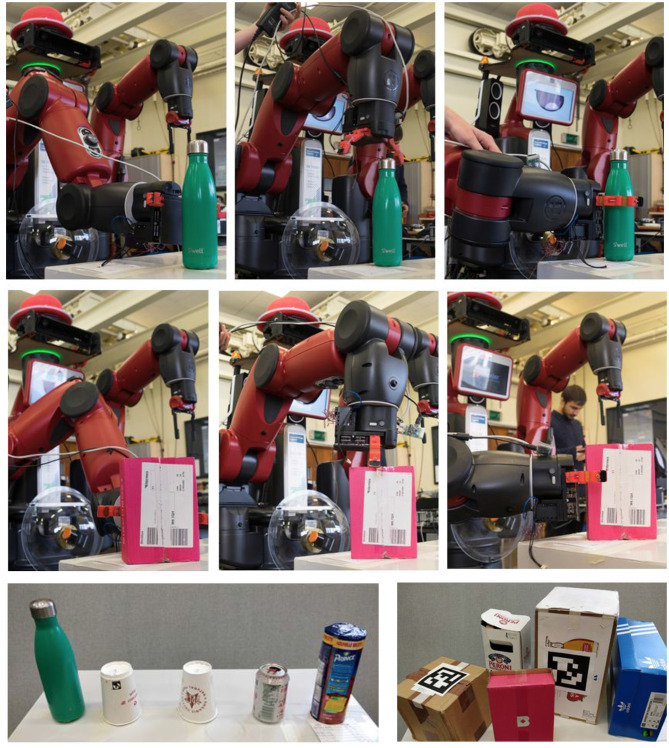
Grasping poses of cylindrical **(top)** and box-shaped **(middle)** objects with the custom-made bio-inspired grippers. From left to right, the grasping poses are front, top and side. **Bottom**: All cylindrical and box-shaped objects used for this challenge. Written consent for using this figure by the person depicted has been obtained.

Figure 12 illustrates the grasping poses of the custom-made bio-inspired grippers for the cylindrical object (top) and the box-shaped object (bottom). The grasping pose for each object depends on three factors:

1. The gripper used for manipulation,
2. The type of object grasped, and
3. The pose of the object.

The rotational orientation of the grasping pose is manually defined by identifying an effective stance to grasp an object of a certain shape. If colliding objects are identified within a specific grasping pose, we allow DE NIRO to choose a different grasping procedure during the operation. Note that a grasping pose will only be considered valid if the model is considered feasible within the physical constraints of the gripper (e.g., if box dimensions are smaller than maximum gripper span).

#### 5.6.2. Tests to Understand the Planning Scene

We made two attempts to validate our vision pipeline. First, we tested robustness with respect to the identification of shape parameters. We validated the consistency of the measurements with regards to the object parameters by placing several example objects in five different poses each and estimating their parameters with RANSAC. We empirically found standard deviations below 5% of the average prediction for each dimension, indicating a rather consistent model dimension estimate.

Next, we tested the robustness of the pipeline by repeatedly estimating the model parameters as returned by the RANSAC algorithm for ten example objects (five boxes with three parameters each and five cylinders with two parameters each), all in five different poses. Then, we compared these to the ground truth object parameters, as physically measured (repeated for 5 trials each time). We observed an average absolute errors for the box parameters as 11.0%, 15.0%, and 17.1% and for the cylinder parameters of 7.4% and 12.8%.

Our results imply an estimation error accurate enough for the cylinders to be grasped successfully at their centers. For the box-shaped objects, the estimation error was too large to produce reliable grasps, as the center location depends significantly on the object parameters, leading to a high probability of failed grasps. Given this limitation, we extended our work with a second pipeline specifically geared toward grasping boxes. We discuss this extensively in section 6.

#### 5.6.3. Tests for Motion Planning and Grasping

We also conducted tests to validate the precision of the motion planning and grasping procedures. We defined the task as placing an example object in varying target zones on a table while using object-dependent grasping poses. We repeated this process and found, given a physically reachable pose, an approximate success rate of 90%, with 10% of the trajectories either as too complex (very erratic movement) or resulting in collisions. In addition, we found that the custom claw-like grippers performed significantly better than the original Baxter pincers. In particular, the custom grippers were more compliant to errors in the estimated pose of the objects.

This overall implementation challenge extended our understanding on how DE NIRO might be able to more intelligently seek out, grasp, and manipulate objects of variable shapes, without knowing their characteristics ahead of time. This was a step beyond our earlier implementation, in which DE NIRO would use automatic processes to try to grasp an object that was in a position it expected. With this extension, DE NIRO was operating more autonomously and more generally. In the next section, we extend our exploration of whether DE NIRO, as a humanoid robot, can show more facility with grasping larger box-shaped objects.

## 6. Implementation: Motion Planning for Dual-Arm Object Manipulation

### 6.1. Overview

The previous two sections demonstrated DE NIRO's ability to operate pick-and-place tasks with just one arm. However, some procedures might benefit from having two arms. Our goal here is to grasp and lift box-shaped objects of potentially varying weights. Like fetching and complex object detection, manipulating larger objects falls into a basket of tasks that are likely to be common in a domestic environment. Expanding DE NIRO's abilities to confront a variety of external environments and act on them appropriately is the aim of this research, and making use of one of DE NIRO's most evident features—its two arms—is a natural next area of research.

For one thing, using two arms offers greater maneuverability. Objects might be very big or unevenly shaped (say, a box without handles), and two touch points might offer more control or balance than one. Similarly, objects might be unstable, requiring repositioning and dynamic rebalancing during the procedure, like a typical peg-in-hole task with one arm positioning the peg and one harm holding the hole (Edsinger and Kemp, 2008). In addition to maneuverability, a closed kinematic chain consisting of two arms can control the stiffness and strength of manipulation in a controlled way. This means they can apply squeezing or twisting forces, or even reshape deformable materials. Tasks like pulling a piece of fabric are not feasible without this additional complexity. Third, dual-arm manipulation more naturally resembles the capabilities of a human. When performing manipulation through teleoperation with an exoskeleton, as discussed in section 3.3, the bimanual capabilities of a user can be more intuitively transferred to the recipient robot (Falck et al., 2019). When applying robots to environments originally intended for humans, such as social assistance robotics, having a human form factor in its manipulation capabilities may be beneficial (Wimböck and Ott, 2012). This is what has motivated recent humanoid flagship robots, such as Boston Dynamics's ATLAS and HANDLE robots (Boston Dynamics, 2019a,b).

As in sections 4 and 5, we subdivide our implementation into component parts:

1. **Vision:** DE NIRO must estimate the target object's pose.
2. **Kinematics:** DE NIRO needs to position its arms appropriately for the target object.
3. **Motion Planning:** DE NIRO must move its arms in such a way as to avoid collisions with itself or any external objects
4. **Arm Trajectory:** DE NIRO needs to actually move its arms and respond to unexpected obstacles along the way.
5. **Grasping:** DE NIRO finally controls and manipulates the target object, accounting for various possible errors.

### 6.2. Vision

**Figure 13:**
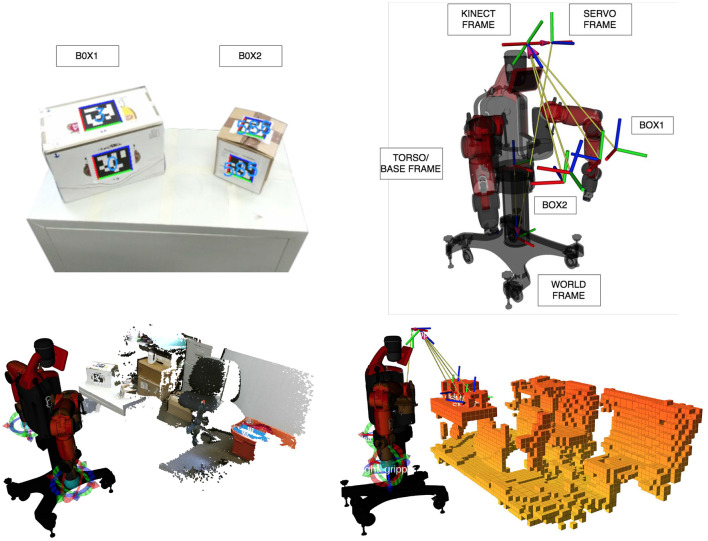
**Top left**: Two boxes with attached AprilTags and their recognized transformation. **Top right**: Transformation chain from the recognized object transformation to the world frame. **Bottom left**: Point cloud as recorded by the Kinect, including RGB information. **Bottom right**: Voxel representation of DE NIRO's surrounding environment, as computed by OctoMap. Occupied voxels have been filled in with orange.

Before grasping a box, DE NIRO must estimate its pose in 3D space. As in section 4, we used fiducial markers, which we knew to yield highly accurate and robust results. The primary disadvantage of fiducial markers is as before: each object that might have to be lifted needs to be labeled ahead of time. Nonetheless, the fiducial markers do allow the robot to accurately estimate the distance to an object, given a proper calibration of the Kinect camera (as discussed in section 5.2.1). For this challenge, we used the AprilTag algorithm together with the AprilTag variety of fiducial markers (Wang and Olson, 2016). We attached these fiducial markers to two box objects, with the recognized poses shown in Figure 13 (top left). Once the pose of the object is estimated, we can calculate its position and orientation relative to other points that are kinematically relevant to DE NIRO, following the transformation chain shown in Figure 13 (top right).

In order to perceive the environment, we used the OctoMap algorithm, a probabilistic mapping framework based on octrees that creates an occupancy map (Hornung et al., 2013). The mapping procedure and resulting voxel representation is illustrated in Figure 13 (bottom). Given the occupancy map, a planner, explained in the subsequent section, can generate an arm trajectory that avoids collisions by circumventing occupied voxels.

### 6.3. Manipulation Kinematics

**Figure 14:**
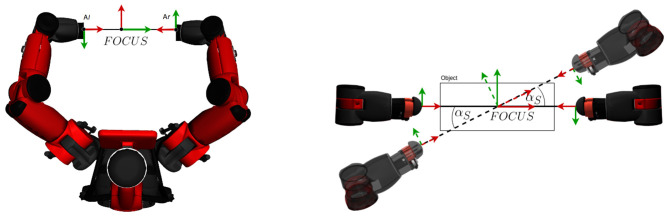
**Left**: The default position of DE NIRO's hands normal to a target object and facing one another. **Right**: The calculation of the center coordinate system in response to a clockwise movement of the object (looking down upon it).

Following the taxonomy of dual-arm manipulation by Kruse (2016), we focus mostly on coordinated, bimanual, symmetric behavior that uses both arms to regulate the internal force and position in order to grasp a box. To do so, we aim at defining a symmetric kinematic pose of the arms, illustrated in Figure 14 (left), with an coordinate system (named “FOCUS”) between two equidistant end-effectors.

By default, both arms face the side of the target object, along its surface normal. This allows for a very intuitive procedure for dual-arm manipulation: if DE NIRO's hands shift an object's position, DE NIRO can simply change the position or rotation of the FOCUS coordinate system to compensate. We demonstrate this motion in Figure 14 (right). Similarly, to grasp or release an object, the distance between the origins of FOCUS and the two end-effector coordinate systems (named “*A*_*l*_” and “*A*_*r*_”) is reduced or increased accordingly.

### 6.4. Motion Planning

**Figure 15:**
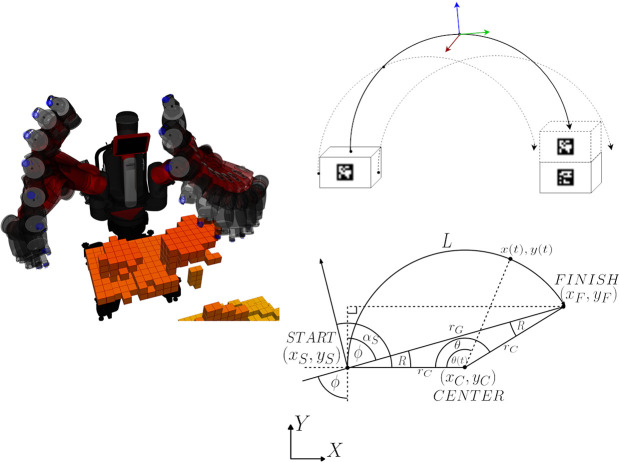
**Left**: Trail of a collision-free motion around occupied voxels (orange) planned with RRTConnect. **Right**: Pick-and-place trajectory inspired by Ackermann steering **(top)** and its geometric description **(bottom)**.

To plan DE NIRO's arm movements, we use MoveIt!, which already has an implemented library of global planners. Most of these planners use a probabilistic roadmap method to iteratively sample from the configuration space, considering any obstacles or occupied space detected during the mapping process in section 6.2 (LaValle, 2006). In order to avoid collisions of the robot arms, we used the Flexible Collision Library (FCL), which uses the Axis-Aligned Bounding Boxes (AABB) method (Pan et al., 2012). We planned a trail of a collision-free motion around occupied voxels with RRTConnect (Kuffner and LaValle, 2000), as illustrated in Figure 15 (left).

In order to do collision checking in real-time, DE NIRO's arm poses must be synchronized with the dynamic OctoMap. To ensure that our clocks were highly synchronized, we connected DE NIRO's Baxter core and the two controlling computers mounted on its back with a UK-wide NTP server pool. By comparing that time in the Baxter core with the incoming Octomap time, we could determine if one was ahead of the other, and if so, by how much. Lastly, we controlled the robot with trajectory waypoint commands through the Joint Trajectory Action Server (JTAS).

### 6.5. Arm Trajectory

Despite determining the kinematics of box manipulation and uninterrupted motion planning, it is still not clear what movement the robot should execute. Our original scenario was a pick-and-place task. Imagined in a context where DE NIRO may need to stack larger objects or move them inside shelves, we define a simple, yet flexible solution to this task inspired by Ackermann steering, in which a car follows a two-dimensional arc (Mitchell et al., 2006). Figure 15 (right) illustrates the desired trajectory intuitively; below, we derive it geometrically in compact form.

Given the definition of angles, distances, and a coordinate system in Figure 15, our objective is to move the box from the starting position (START) with coordinates (*x*_*S*_, *y*_*S*_) to the goal position (FINISH) with coordinates (*x*_*F*_, *y*_*F*_) and describe its trajectory with respect to time (*x*(*t*), *y*(*t*)). Considering this motion, we can see that an isosceles triangle between the CENTER position (*x*_*C*_,*y*_*C*_), START and FINISH is formed. From the trigonometric equations, it holds that

(1) $$\begin{array}{l} sin\left(\theta \right)=2sin\left(\frac{\theta }{2}\right)cos\left(\frac{\theta }{2}\right) \end{array}$$

By considering the two right-angled triangles within the isosceles triangle, we get

(2) $$\begin{array}{l} 2sin\left(\frac{\theta }{2}\right)=\frac{2r_{G}}{2r_{C}} \end{array}$$

and

(3) $$\begin{array}{l} cos\left(\frac{\theta }{2}\right)=\frac{\sqrt{{r_{C}}^{2}-{\left(\frac{r_{G}}{2}\right)}^{2}}}{r_{C}}=sin\left(R\right) \end{array}$$

Substituting in Equation (1) with Equations (2) and (3), we get

(4) $$\begin{array}{l} \frac{\text{sin}\left(\theta \right)}{r_{G}}=\frac{\text{sin}\left(R\right)}{r_{C}} \end{array}$$

Furthermore, let ϕ be the angle between the connecting line of START and FINISH and the tangent of the arc motion in START. Knowing that ϕ and R are complementary angles, we get

(5) $$\begin{array}{l} \phi +R=\frac{\pi }{2} \end{array}$$

and

(6) $$\begin{array}{l} \theta +2R=\pi \end{array}$$

therefore

(7) $$\begin{array}{l} \theta +2\left(\frac{\pi }{2}-\phi \right)=\pi \Rightarrow \theta =2\phi \end{array}$$

and by substituting θ and *R* in Equation (4) with these results, we get

(8) $$\begin{array}{l} r_{C}=r_{G}\frac{\text{sin}\left(\frac{\pi }{2}-\phi \right)}{\text{sin}\left(2\phi \right)} \end{array}$$

Using the cofunction and double angle identities

(9) $$\begin{array}{l} \text{sin}\left(\frac{\pi }{2}-\phi \right)=\text{cos}\left(\phi \right) \end{array}$$

(10) $$\begin{array}{l} \text{sin}\left(2\phi \right)=2\text{ sin}\left(\phi \right)\text{cos}\left(\phi \right) \end{array}$$

and substituting these into Equation (7) yields

(11) $$\begin{array}{l} r_{C}=r_{G}\frac{\text{cos}\left(\phi \right)}{2\text{ sin}\left(\phi \right)\text{cos}\left(\phi \right)}=\frac{r_{G}}{2\text{ sin}\left(\phi \right)} \end{array}$$

We can now find the total distance L travelled in the arc trajectory from START to FINISH as follows (note that θ is measured in radians)

(12) $$\begin{array}{l} L=r_{C}\theta \end{array}$$

(13) $$\begin{array}{l} L=r_{C}\left(2\phi \right),\text{since }\theta =2\phi \end{array}$$

(14) $$\begin{array}{l} L=\frac{r_{G}\phi }{\text{sin}\left(\phi \right)},\text{since }r_{C}=\frac{rG}{2\text{ sin}\left(\phi \right)} \end{array}$$

Let us now consider the time delta (*t*_*F*_ − *t*_*S*_) it takes to traverse L from START to FINISH

(15) $$\begin{array}{l} t_{F}-t_{S}=\frac{L}{v},\text{where }v\text{ is velocity}. \end{array}$$

θ(*t*), the angle between the horizontal and the connecting line between CENTER and the current position *x*(*t*), *y*(*t*), changes with constant factor ω

(16) $$\begin{array}{l} \omega =\frac{\theta }{\left(t_{F}-t_{S}\right)} \end{array}$$

We can therefore define θ(*t*) as

(17) $$\begin{array}{l} \theta \left(t\right)=\omega \cdot \left(t-t_{S}\right) \end{array}$$

In our case, α_*S*_, the original orientation of the box itself, stays unchanged throughout the trajectory and can only be set at the start or the end of the movement.

Finally, we return to our initial goal of describing the arc trajectory coordinates x(t), y(t) with respect to time. We have the the CENTER coordinates *x*_*C*_ and *y*_*C*_ as

(18) $$\begin{array}{l} x_{C}=x_{S}+r_{C} \end{array}$$

(19) $$\begin{array}{l} y_{C}=y_{S} \end{array}$$

Thus, for a clockwise turn based on a given radius, we can substitute Equation (16) as follows to find the arc trajectory coordinates as

(20) $$\begin{array}{l} x\left(t\right)=x_{C}-r_{C\;}\text{cos}\left(\theta \left(t\right)\right) \end{array}$$

(21) $$\begin{array}{l} y\left(t\right)=y_{C}+r_{C\;}\text{sin}\left(\theta \left(t\right)\right) \end{array}$$

In practice, we compute x(t), y(t) with regular time shift from Equations (23) and (24) and iteratively change the position of the FOCUS coordinate system origin, as discussed in section 6.3. While this type of arc trajectory worked reliably during this challenge, a more sophisticated solution could take into account speed and acceleration constraints by, for example, calculating the trajectory based on smooth, higher-dimensional polynomials along defined waypoints.

### 6.6. Grasping

The symmetric, coordinated dual-arm procedure allows DE NIRO to control and manipulate target objects with a higher-level control scheme. This control scheme needs to be robust to errors and undesirable behavior and act upon errors with exception loops. Examples of such exceptional behaviors covered in our implementation are:

1. Failure to retrieve a feasible kinematic solution from the Baxter inverse kinematics solver,
2. Unsynchronized transformation lookups that incorrectly extrapolate into the future or review the past,
3. A non-existent object transform, because the object is obstructed or outside of the camera frame, and
4. Lost network packets due to network connection issues.

For each of these and other cases, we define exception handling procedures to elegantly overcome error scenarios with automatic response mechanisms during manipulation.

Because arm trajectories can be particularly complex, we added extra validation procedures into the inverse kinematics solver solutions. These check for whether the obtained joint angle solutions are reachable and not too distant from the current position. Given the current Baxter pose and the retrieved kinematic solution, the procedure calculates the Euclidean distance between the current angles of the left and right arm and the proposed angles of the inverse kinematics solver. It requires those distances to be below a user-defined and tuned threshold for the solution to be accepted and executed. This validation procedure avoids motions that might be too large between two sequential steps of the control loop; that could potentially be harmful to the robot hardware or unexpected for the interacting user.

### 6.7. Discussion

We validated the dual-arm manipulation procedure discussed and implemented above with both qualitative and quantitative test scenarios that demonstrate its general applicability. We focused particularly on evaluating global motion planners with respect to their robustness. We measured robustness with a motion success rate, defined as whether the target point B was reached from the initial point A under reasonable time constraints.

**Figure 16:**
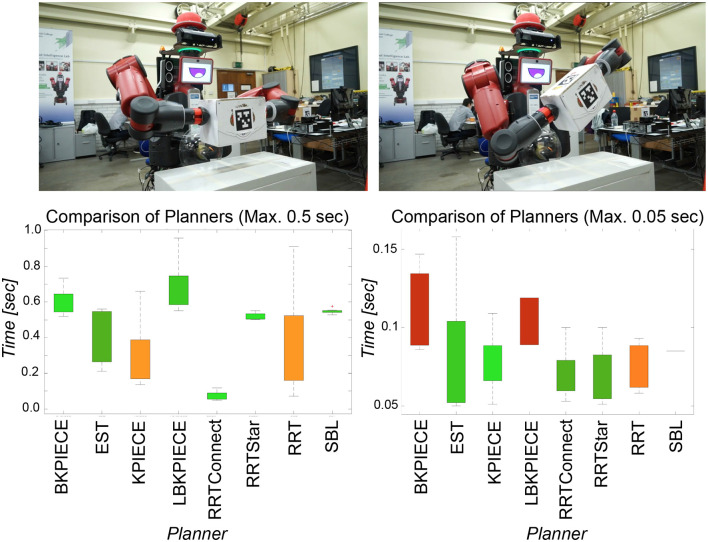
**Top**: Qualitative attempt to manipulate a box with two arms. **Bottom**: Quantitative results with various motion planners and a maximum processing time (soft constraint) of 0.5 seconds **(left)** and 0.05 seconds **(right)**, respectively.

Figure 16 (top) illustrates DE NIRO's box grasping capabilities and the kinematics of rotating the box in action. While the kinematic solution turned out to work quickly and reliably, motion planning is intrinsically slower as further restrictions imposed by a dynamic environment must be taken into account. This real-world scenario in a domestic environment requires significantly more effort compared to a fixed, static manufacturing environment with clearly defined barriers and contact points, as the robot needs to iteratively plan the motion. This is a distinct feature in our setting.

For this reason, we analyze different types of motion planners for our dual-arm box manipulation task in Figure 16 (bottom). In particular, we are interested in evaluating global motion planners with soft constraints—which can be violated if the solution is far off—against a maximum processing time of 0.5 seconds and 0.05 seconds, in order to achieve natural reactions to a user. The boxplots illustrate the distribution of the processing time required to come up with a valid solution. The color (traffic-light scale) of the boxplots indicates the success rate of the executed trajectory while holding a box across 10 runs per planner.

In Figure 16 (bottom left), red indicates frequently unsuccessful; orange, moderately successful; and green, always successful trajectories. We conclude that given a reasonable, yet short processing time of 0.5 seconds, most of the planners (except KPIECE and RRT) find a successful solution. Furthermore, some algorithms, such as RRT, vary strongly in their processing time, while others, such as RRT^*^, show a low variation. If the processing time is further reduced by a factor of ten (bottom right), many algorithms become unreliable. KPIECE, however, performed most stably under these tighter soft constraints. Regardless of the constraints, RRTConnect performs reliably with a small, low-variant processing time. This is therefore the algorithm we used as a solution to this challenge.

## 7. Conclusion

In this paper^1^, we presented the design and implementation of Robot DE NIRO to support adaptive, complex manipulation behaviors that may be common in a domestic environment. Such work may make DE NIRO particularly useful in support of geriatric nurses who interact with care recipients.

Currently, DE NIRO is limited in various ways and we list a few of these in the following. First, its design is non-holonomic, limited only to forward, backward, translational, and rotational movement. DE NIRO cannot move sideways without turning first. Second, with a maximum payload of 2.2 kg per arm, DE NIRO is limited to relatively lightweight tasks. It is, for example, incapable of lifting a human body. Third, due to limited sensor capabilities in the current design, we have constrained DE NIRO to trajectories using forward motion. This can result in the robot getting stuck in corners.

Despite this, DE NIRO can go much further. DE NIRO would benefit from concurrency across its components. Rather than primarily one sensor active at a time, its abilities would increase by being able to move and grasp together, for example. It would also help to have more robust localization that does not require predefined mapping (Bloesch et al., 2018) and semantically richer spatial information for a more complex decision making (Landgraf et al., 2020). We noted earlier that during navigation, DE NIRO freezes if it believes it is colliding with something and that it has no way to plan a trajectory that does not entail further collisions. DE NIRO could be made more autonomous and more intelligent by more accurately classifying collision types, having a better sense of its own shape (not just its pose), and avoiding deadlock situations (Kormushev and Ahmadzadeh, 2016). Dynamic objects in these environments are usually humans. DE NIRO currently detects humans by matching incoming images of faces to saved images, but it does not have a more general understanding of the human pose and form. Were it to do so, it would be a more complete assistant, perhaps even being able to learn skills after human demonstration (Ahmadzadeh et al., 2013). Our work on object recognition and grasping made clear that further improvements here were possible, too. Point cloud-based object detection, for example, may provide more elegant grasping techniques (Rusu et al., 2008; Gajewski et al., 2018). In addition, we have not yet combined the extensions we researched in object detection and dual-arm object manipulation with the original fetch task. Another fruitful next area of research would be to integrate all these capabilities and then have a wide population of humans actually interact with DE NIRO in a live setting.

All this being said, the work we have accomplished here shows that DE NIRO can be used to provide reliable, efficient support to tasks requiring frequent, natural interaction with humans. Our goal was to build these capabilities into DE NIRO itself, but also to share this model openly to other researchers. Should any others wish to quickly build a robot with increasingly abstract abilities, we hope they might be able to use some or all of our solutions in their efforts.

## Data Availability Statement

The original contributions presented in the study are included in the article/supplementary materials, further inquiries can be directed to the corresponding authors.

## Ethics Statement

Written informed consent was obtained from the individual(s) for the publication of any potentially identifiable images or data included in this article.

## Author Contributions

FF and SD were the main editors of the paper. SD, FF, JL, KR, and NS contributed to section 4. MT contributed to section 5. GN contributed to section 6. All other sections are joint efforts by the authors, with RS, KW, and PK contributing particularly to the hardware and software design of DE NIRO in section 3.

## Conflict of Interest

The authors declare that the research was conducted in the absence of any commercial or financial relationships that could be construed as a potential conflict of interest.

## Footnotes

^1^We have made our code base open source, provided extensive documentation, and published a video illustrating some of DE NIRO's current capabilities here: https://www.imperial.ac.uk/robot-intelligence/software/
